# Key incidents identified by the Royal College of Pathologists of Australasia Quality Assurance Programs Key Incident Monitoring and Management EQA program

**DOI:** 10.11613/BM.2026.010701

**Published:** 2025-12-15

**Authors:** Anushka Jayanetti, David Roxby, Tony Badrick

**Affiliations:** 1 Royal College of Pathologists of Australasia Quality Assurance Programs, Sydney, Australia; 2 Molecular Medicine and Pathology, College of Medicine and Public Health, Flinders Medical Centre, Adelaide, Australia

**Keywords:** preanalytical phase, incident reporting, risks management, quality improvements, Benchmarking

## Abstract

**Introduction:**

The Royal College of Pathologists of Australasia Quality Assurance Programs (RCPAQAP) Key Incident Monitoring and Management EQA Program (KIMMS) aims to monitor the laboratory quality system’s pre- and post-analytical phases. The purpose of this paper is to describe the most common incidents from 2024.

**Materials and methods:**

The KIMMS program has four surveys a year, collecting data from the previous three months, with preanalytical and postanalytical incident reporting of 35 incident types. Participants are asked to capture the number of episodes and the number of incidents per quarter of the year.

**Results:**

The four 2024 surveys received an average of 111 responses, with 55,329,998 episodes recorded and 1,496,708 incidents identified. The findings from the 2024 program are that the incident “No specimen received” appears to have the highest 80th percentile across the patient sources. The commonest site of error is the Emergency Department (ED), with an 80th percentile overall.

**Conclusions:**

The KIMMS data provide valuable, regular and reproducible benchmarking data for the pre- and post-analytical phases of the total testing cycle.

## Graphical abstract

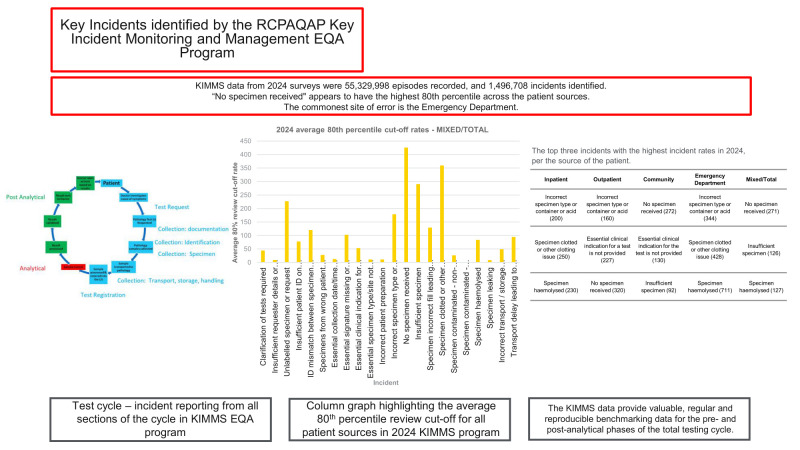

## Highlights

• Key Incident Monitoring and Management EQA Program data from 2024 surveys was 55,329,998 episodes recorded, and 1,496,708 incidents identified

## Introduction

Pre- and postanalytical errors represent most errors in the total testing cycle leading to increased risk of misdiagnosis and treatment of patients (*1*-*3*). Identification, phlebotomy, collection tube and sample preservation and transport related errors are common sources of error, particularly when the specimen collection staff are not laboratory-controlled (*4*). Postanalytical errors mainly involve delayed or incomplete delivery of results to referring doctors. Despite this, few benchmarking EQA programs are available for medical laboratories to assess these error rates objectively (*5*, *6*). Indicators of the extra-analytical phases of the Total Testing Process (TTP) have been developed in several countries, such as Australia and New Zealand, The US Brazil, and Spain/Catalonia, and other surveys and programs have been promoted in the UK, China and Croatia (*7*-*14*). In 2008, the IFCC launched a Working Group named “Laboratory Errors and Patient Safety” (WG: LEPS) to identify Quality Indicators (QIs) and related quality specifications that (i) produce benchmarks from comparing laboratories, (ii) promote error reduction and (iii) increase patient safety. The IFCC has developed QIs, which laboratories in several countries have evaluated, and the WG: LEPS has reported preliminary results (*15*-*17*).

To reduce pre- and post-analytical error, laboratories need to focus on these phases of the total testing cycle with relevant QIs and benchmarks from external sources (*18*-*20*). As with any form of benchmarking, laboratories must collect and track their QIs and then compare them against the relevant peer group. Initially, this ensures they collect the same data as their peers, then compare their performance. If there are significant differences after data collection error has been eliminated, then a review of the process is warranted. To ensure there is confidence in this comparison, a sufficiently large peer group collecting the same QIs needs to be available, and the data collection needs to be regular enough to detect trends (*21*).

But collecting QI data in pathology can be tedious. Benchmarking processes are far more difficult than standard analytical EQA because measurement may be complex and not standardised, data collection may not be easy, and targets are unavailable. As with many benchmarking exercises, there have been problems with the definitions and robustness of the QIs. Key Incident Monitoring and Management EQA Program has tried to ensure that participants collect and submit the same data for each of the QIs. While progress has been made in this direction, the data is still not always homogeneous. One of the aims of the KIMMS program is to produce a list of key incidents that should be tracked by an LIS. Also, changing a process is more difficult than identifying an error in an assay system.

The Royal College of Pathologists of Australasia Quality Assurance Programs (RCPAQAP) Key Incident Monitoring and Management EQA Program (KIMMS) started in 2011 and aims to monitor the pre- and post-analytical phase of the laboratory quality system (*7*). Key Incident Monitoring and Management EQA Program measures risks from incidents recorded by participant laboratories and compares these to those of peer organisations. Incidents and risks are related but not equal (*22*). The program assesses risk based on the consequences to the patient, the probability that the event will occur, and how easy it is to detect the incident. A reduction in probability or an increase in detectability can reduce the risk. A two-phase risk calculation is used in KIMMS, where a harm factor equals consequences multiplied by likelihood (*21*). The risk score equals harm times detectability (*23*).

As an example of this risk score process, we present this case where the overall performance summary highlights any “incident” that recorded a rate greater than 80% of all participants. This is presented in a table, which includes columns for the consequence, probability and detectability rating (Tables 1-3). The KIMMS Advisory Committee has pre-determined the consequence rating, so this field is pre-populated.

**Table 1:** Example risk score table from the KIMMS report

**Parameter out of allowable limits**	**Consequence rating**	**Probability rating**	**Detectability rating**	**Risk score**	**Organisational response**
Inpatient - hemolysis rate = 400	2	5	1	10	Example: Although this is a low-risk incident, high compared to peers and to our collectors (0.2). Meeting with hospital organised 7/10/22

**Table 2:** KIMMS consequence scale

**Scale**	**Name**	**Definition**
1	Negligible/Minimal	Minimal, delay, inconvenience
2	Marginal/Minor	Recollect required
3	Significant/Moderate	Delayed management (non-malignant) and/or medical treatment
4	Serious/Major	Delayed diagnosis (malignant) and/or surgical treatment
5	Critical/Catastrophic	Serious harm to multiple patients and/or patient death

**Table 3:** KIMMS probability scale

**Scale**	**Name**	**Example Definition**
1	Rare	< 1/year
2	Unlikely	1 *per* year
3	Occasional	1 *per* month
4	Likely	1 *per* week
5	Frequent	1 *per* day or more

### Results review

It is recommended that participants review their results by applying the probability and detectability rating to determine the risk score (Tables 2-4). The risk score is calculated by multiplying the three ratings (consequence rating x probability rating x detectability rating). The risk score can be used as a guide to organisational risk; from that, a decision is made as to the next step(s).

**Table 4:** KIMMS detectability scale

**Scale**	**Name**	**Definition**
1	Detected	> 95%
2	Most detected	75-95%
3	Half detected	25-75%
4	Most not detected	< 5-25%
5	Not detected	< 5%

In the above example, the consequence, probability and detectability rating give a risk score of 10. This would be considered low risk. Apart from flagging that the risk is higher than other participants’ inpatient collections, it is unlikely that any further immediate action is required. This incident should be monitored closely to ensure it does not deteriorate.

### Risk score

The score is calculated by multiplying the consequences (Table 1) x probability (Table 2) x detectability (Table 3).

The purpose of this paper is to report the most common incidents that have been detected in the 2024 KIMMS program.

## Materials and methods

The KIMMS program has four surveys a year, collecting data from the previous three months, with preanalytical and postanalytical incident reporting of 35 incident types that have been pre-determined by the RCPAQAP KIMMS Advisory Committee. Data is submitted *via* the RCPAQAP myQAP portal, where result entry instructions are included. Participants are asked to capture the number of episodes and the number of incidents *per* quarter of the year.

Data is then analysed using RCPAQAP in-house data analysis software, where each specific incident reported to KIMMS is calculated *per* 100,000 episodes (number of incidents divided by the number of episodes, multiplied by 100,000), known as the rate. Incident rates are compared to peer groups and flagged red if outside the best performers, which is set at 80% of all results. This summary will focus on 2024 data.

A single request usually covers an episode and may contain one or more samples. It may include more than one request, but the samples are collected simultaneously, *i.e.*, samples received together in a bag with a request form equal one episode.

Participants range from small to large pathology laboratories, where size is determined by the number of episodes *per* quarter (small: < 100,000, medium: 100,000-500,000, large: > 500,000). This data is also collected for each submission. The participants include a mix of public and private organisations from across Australia and a couple of international sites.

### Patient sources

The KIMMS program was renewed in 2023 to allow for further comparison by separating data into categories such as the patient source (Inpatient/Outpatient/Community/Emergency Department (ED)).

The Inpatient patient source refers to pathology testing performed on an admitted hospital patient (public or private hospital). The Outpatient source includes pathology requests and specimens that are collected while a patient attends a clinic run by a hospital. This includes day patients and cancer treatment centres. The Emergency Department patient source refers to pathology testing performed on a patient attending an Emergency Department (ED)/Emergency Room (ER)/Accident and Emergency (A&E)/Casualty ward/department. The community patient source is for when the pathology request is generated anywhere, but the specimens are collected in settings other than the previously mentioned patient sources. This includes doctors’ surgeries, designated collection centres (even within a hospital), nursing homes, home visits, and workplace collections.

The KIMMS program also offers a Mixed patient source option for those organisations that are unable to differentiate their data between patient sources. In the final calculations, these are analysed with a total of all results.

## Results

The four 2024 surveys received an average of 111 responses, with 55,329,998 episodes recorded and 1,496,708 incidents identified.

Table 5 summarises the total episodes and incidents reported *per* survey. Table 6 summarises the key results for the top three incidents from all sites, and Table 7 shows the key results *per* size of organisation. Tables 8-10 display the average 80th percentile cut-off rates and standard deviations (SDs) *per* patient source and the Mixed/Total category for the Test Request/Collection incident categories, as well as the Test Registration/Analytical/Postanalytical incident categories from the four 2024 KIMMS surveys.

**Table 5:** The total number of episodes and incidents reported in each 2024 KIMMS survey

	**Total episodes**	**Total incidents**
Survey 1	11,930,241	274,277
Survey 2	13,123,033	295,720
Survey 3	14,832,081	484,514
Survey 4	15,444,643	442,197

**Table 6:** The top three incidents with the highest incident rates in 2024, *per* the source of the patient

**Inpatient**	**Outpatient**	**Community**	**Emergency Department**	**Mixed/Total**
Incorrect specimen type or container or acid (200)	Incorrect specimen type or container or acid (160)	No specimen received (272)	Incorrect specimen type or container or acid (344)	No specimen received (271)
Specimen clotted or other clotting issue (250)	Essential clinical indication for a test is not provided (227)	Essential clinical indication for the test is not provided (130)	Specimen clotted or other clotting issue (428)	Insufficient specimen (126)
Specimen hemolysed (230)	No specimen received (320)	Insufficient specimen (92)	Specimen hemolysed (711)	Specimen hemolysed (127)
Values in brackets represent the rates.				

**Table 7:** The top three incidents with the highest incident rates in 2024, *per* the size of the organisation (episodes *per* quarter)

**Small organisation** **(< 100,000 episodes)** **N = 89**	**Medium organisation (100,000-500,000 episodes)** **N = 13**	**Large organisation** **(> 500,000 episodes)** **N = 9**
Essential collection date/time not provided or discrepant between specimen and request (401)	No specimen received (407)	No specimen received (528)
Incorrect specimen type or container or acid (589)	Insufficient specimen (322)	Essential clinical indication for the test is not provided (331)
Specimen clotted or other clotting issue (466)	Specimen clotted or other clotting issue (411)	Specimen hemolysed (280)
The “N” value refers to the average number of submissions *per* survey in 2024 that selected that particular size of organisation.		

**Table 8:** Average 80th percentile cut-off rates *per* patient source for the Test Request/Collection incident categories from the four 2024 KIMMS surveys

	**Inpatient**	**Outpatient**	**Community**	**ED**
Clarification of tests required	109.4	140.2	0.9	263.6
Insufficient requester details or signature missing	3.2	0.0	7.5	8.1
Unlabelled specimen or request	210.4	247.5	128.6	272.2
Insufficient patient ID on specimen and/or request	113.4	91.3	85.2	100.4
ID mismatch between specimen and/or request	161.8	100.9	61.9	200.6
Specimens from wrong patient (WSIT)	35.5	24.1	1.6	44.8
Essential collection date/time not provided or discrepant between specimen and request	14.1	9.3	2.3	34.3
Essential signature missing or discrepant on transfusion sample and/or request	113.6	83.0	17.9	185.6
Essential clinical indication for test not provided	41.7	78.8	40.3	18.5
Essential specimen type/site not provided	6.0	0.0	10.9	3.3
Incorrect patient preparation	8.2	0.0	12.2	9.0
Incorrect specimen type or container or acid	228.7	158.0	75.5	208.3
No specimen received	421.4	521.7	378.5	589.4
Insufficient specimen	335.5	252.6	147.8	458.2
Specimen incorrect fill leading to incorrect specimen:additive ratio	153.7	88.8	42.0	199.3
Specimen clotted or other clotting issue	385.4	161.6	51.1	679.7
Specimen contaminated - non-microbiological	40.7	17.8	4.6	33.6
Specimen contaminated - microbiological	0.0	0.0	0.1	1.8
Specimen hemolysed	75.1	37.1	44.1	416.0
Specimen leaking	0.0	0.0	3.8	13.1
Incorrect transport / storage temperature / handling	46.5	65.2	50.9	33.0
Transport delay leading to specimen being too old to test	109.7	135.1	45.8	82.6

**Table 9:** Average 80th percentile cut-off rates for the Mixed/Total patient source for the Test Request/Collection incident categories from the four 2024 KIMMS surveys

	**Mixed/Total**
Clarification of tests required	44.3
Insufficient requester details or signature missing	9.1
Unlabelled specimen or request	227.2
Insufficient patient ID on specimen and/or request	78.0
ID mismatch between specimen and/or request	120.5
Specimens from wrong patient (WSIT)	28.0
Essential collection date/time not provided or discrepant between specimen and request	12.9
Essential signature missing or discrepant on transfusion sample and/or request	102.4
Essential clinical indication for test not provided	53.1
Essential specimen type/site not provided	11.1
Incorrect patient preparation	11.0
Incorrect specimen type or container or acid	178.7
No specimen received	425.7
Insufficient specimen	290.4
Specimen incorrect fill leading to incorrect specimen: additive ratio	129.4
Specimen clotted or other clotting issue	359.8
Specimen contaminated - non-microbiological	26.7
Specimen contaminated - microbiological	0.0
Specimen hanalytemolysed	83.9
Specimen leaking	8.6
Incorrect transport / storage temperature / handling	49.4
Transport delay leading to specimen being too old to test	94.9

**Table 10:** Average 80th percentile cut-off rates for all results for the Test Registration/Analytical/Postanalytical incident categories from the four 2024 KIMMS surveys

	**Total**
Incorrect unique specimen identifier	8.4
Patient ID - wrong patient	7.1
Errors in transcription of patient demographic information	31.7
Incorrect or missed tests	183.1
Incorrect or missing specimen type, site, collection time	26.0
Incorrect requesting or copy doctor	131.7
Internal laboratory process incident - unfixable	43.0
Within laboratory ID error	6.4
Intra or inter-laboratory specimen lost or misplaced - irreplaceable	0.2
Intra or inter-laboratory specimen lost or misplaced - replaceable	10.0
Failures of clinical handover high-risk (critical) results	0.1
Failures of clinical handover non-critical results	0.1
Amended reports, significant patient impact	47.7

**Figure 1:**
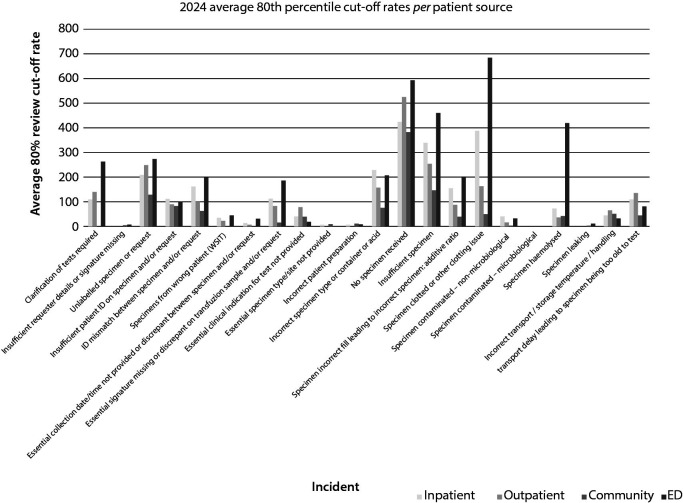
Average 80th percentile cut-off rates *per* patient source for the Test Request/Collection incident categories from the four 2024 KIMMS surveys.

**Figure 2:**
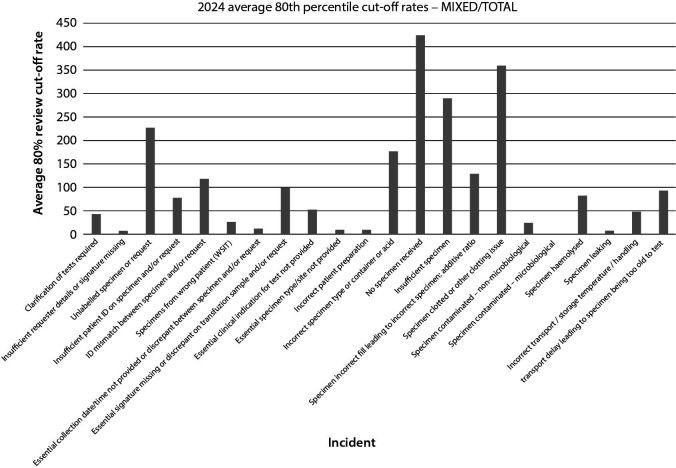
Average 80th percentile cut-off rates for the Mixed/Total patient source for the Test Request/Collection incident categories from the four 2024 KIMMS surveys.

**Figure 3:**
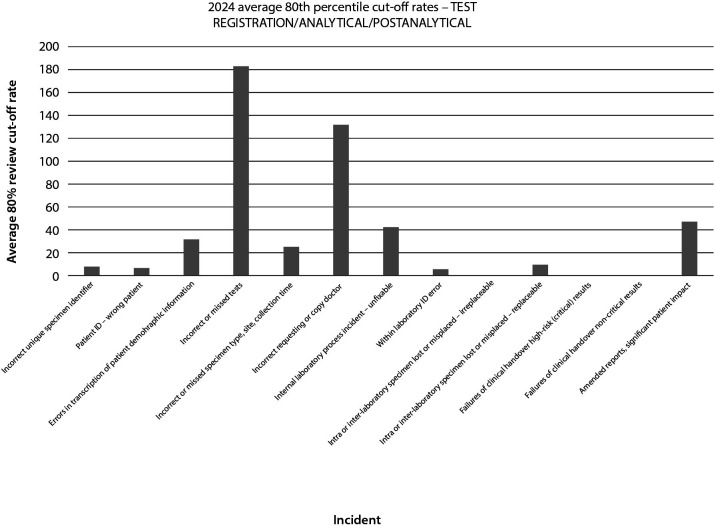
Average 80th percentile cut-off rates for all results for the Test Registration/Analytical/Post-Analytical incident categories from the four 2024 KIMMS surveys.

Figures 1-3 are bar graphs highlighting the average 80th percentile cut-off rates *per* patient source and the Mixed/Total category for the Test Request/Collection incident categories, as well as the Test Registration/Analytical/Postanalytical incident categories from the four 2024 KIMMS surveys.

## Discussion

The findings from the 2024 program are that the incident “No specimen received” appears to have the highest 80th percentile across the patient sources. The cost of a recollection is significant, both in organisational cost and patient time (*24*). More importantly, it is an error that can be reduced once identified. Suggested mechanisms to reduce this incident would be computer-generated, or manual checklists of collection tubes required for a given set of requested tests.

The commonest site of error is the ED, with an 80th percentile overall. The primary cause is the non-professional collector and the collection process (*4*, *25*-*27*). The solution could include better training of nursing and medical staff in the ED, though this has had poor long-term effects (*25*). The issue may be costly enough for the organisation to place phlebotomists in ED (*28*).

The 80th percentiles are consistent for all incidents throughout the year, meaning the occurrence of incidents is also consistent, validating the data collection process. The 80th percentiles are greater overall for incidents that fall under the Collection incident category, such as “No specimen received”, “Insufficient specimen”, “Specimen clotted” and “Specimen hemolysed”. Sites that perform greater than the 80th percentile have the incidents flagged for review. It is then up to the participants to calculate the risk of the incident to their organisation by referring to our KIMMS Matrix Table (https://rcpaqap.com.au/kimms-matrix-table/), which comprises consequence, probability and detectability. Specific incidences, such as WSIT, may have lower rates reported than the actual values, as the detectability of such incidences is low. This is why in the KIMMS risk matrix table, a higher value for detectability can be assigned to increase the overall risk score and severity of these incidents.

The KIMMS program has been operating since 2011 with several improvements (*7*, *21*, *23*). Significant findings and trends have been separately published (*21*, *29*). This experience of participants reporting using standardised definitions of incidents has led to consistent data collections as can be seen from year-on-year results. It has taken some years for this to occur, as there is much variation in what laboratories call episodes and what their capability is to accurately collect these data. The QIs used are relevant to Australian laboratory practice and share commonality with the IFCC set. It is difficult to compare results from different publications as many are one-off surveys rather than a system of regular data collection.

The limitations of this program’s data collection process include some manual data collection processes and likely missing data for some QIs, such as WSIT, because the laboratory is unable to identify these errors (*30*).

In conclusion, the KIMMS data provide valuable, regular and reproducible benchmarking data for the pre- and post-analytical phases of the total testing cycle. The incidents represent areas where the risk to patients can be reduced.

## Data Availability

All data generated and analyzed in the presented study are included in this published article.

## Appendix

**Table 11:** All incidents measured in the KIMMS program and explanation of each incident

**Categories**	**Incident**	**Explanation of incident**
Test request	Clarification of tests required	Collector or laboratory required to contact requestor to confirm test(s) requested as information not clear or illegible on request
	Insufficient requester details or signature missing	This occurs when the information supplied is not sufficient to know which doctor should receive the report, or the request is not signed when a signature is required
	Unlabelled specimen and/or request	This is when sample or request has no patient identifiers
Collection: Identification	Insufficient patient ID specimen and/or request	Minimum identifiers are full name and DOB. Three identifiers may be required *e.g.*, transfusion samples
	ID mismatch between specimen and/or request	ID on specimen label does not match accompanying request form or electronic order
	Specimen from wrong patient (WSIT)	Labelling of specimen and request are concordant, but the actual sample in the container is from a different patient
	Essential collection date and/or time not provided or discrepant between specimen and request	Specimen and form must align especially essential for a Transfusion request
Collection: Documentation	Essential signature missing or discrepant on transfusion sample and/or request	This refers to collector and patient signature
	Essential clinical indication for test not provided	Collector fails to record the clinical indication for the test that is required by the laboratory to aid in the interpretation of results
	Essential specimen type/site not provided	Specimen type and/or site not provided when information is required to aid in appropriate testing and interpretation of results by the laboratory
	Incorrect patient preparation	Patient not fasting, medication taken instead of being withheld, preparation procedure for test not followed
	Incorrect specimen type or container or acid	Incorrect specimen type collected/submitted for test request
	No specimen received	No sample received to do the test
	Insufficient specimen	Insufficient specimen to do any or all tests requested
Collection: Specimen	Specimen incorrect fill leading to incorrect specimen: additive ratio	Under or overfilled Citrate (COAG) tubes
	Specimen clotted or other clotting issue	Most commonly EDTA tubes, but any tube with anti-coagulant may be clotted
	Specimen contaminated - non microbiological	*E.g.* diluted from IV drip; or order of draw incorrect
	Specimen contaminated - microbiological	Contaminated microbiology samples that require a recollection
	Specimen hemolysed	Hemolysis should be counted if the sample is deemed unsuitable for one or more tests due to the level of hemolysis
	Specimen leaking	Any leakage should be recorded, even if there is still sufficient specimen to do the test
Collection: Transport, storage and handling	Incorrect transport/storage temperature/handling	Stored and/or transported at wrong temperature or handling not in accordance with documented procedure
	Transport delay leading to specimen being too old to test	Any delay in transport
	Incorrect unique specimen identifier	Also known as episode; laboratory or accession number. It is the unique identifier for that specimen from that patient collected/submitted on that date/time
	Patient ID wrong patient	This occurs when the wrong patient is chosen at data entry from a list of patients with the same, or similar name. Request associated to incorrect patient demographics
Test registration	Error in transcription of patient demographic information	This occurs when information is incorrectly entered from a request. Registration data manually entered
	Incorrect or missed tests	Test on request form not added at registration or incorrect test added
	Incorrect or missing specimen type, site, collection time	This is when the information is available on the request form or electronic order but is not entered into the LIS
	Incorrect requesting or copy doctor	Referring clinician or copy to clinician not registered to receive copy of the report
	Internal laboratory process incident - unfixable	This refers to any incident that happens after the sample has arrived at the laboratory that is not directly related to performing the test
Analytical	Within laboratory ID error	Error transferring patient Identification within the laboratory
	Intra or inter-laboratory specimen lost or misplaced - irreplaceable	Specimens lost during intra and inter-laboratory transfer that are irreplaceable
	Intra or inter-laboratory specimen lost or misplaced - replaceable	Specimens lost during intra and inter-laboratory transfer that can be recollected
	Failure of clinical handover high-risk (critical) results	Failure to communicate high-risk results to the relevant party within agreed guidelines as documented within your organisation
Postanalytical	Failure of clinical handover non-critical results	Requests to re-send copy of report due to non-receipt by the requestor, including referring laboratories
	Amended report. Significant patient impact	This is amended report due to a diagnostic error or incorrect result being released
DOB - date of birth. LIS - laboratory information system.		
